# 

**Published:** 1874-11

**Authors:** 


### IMPORTANT TO SUBSCRIBERS.

WE are constantly in receipt of letters from parties who state that they have subscribed for the JOURNAL OF HEALTH, and have failed to receive it. We wish it to be distinctly understood that no subscription to the JOURNAL is valid unless made to the Publishers, who alone have power to contract for its regular delivery. If money is sent to any other party, we do not hold ourselves in any way responsible for it. We invariably enclose a receipt, written upon our printed blank bill-heads, to every subscriber, in the first number after the money is received. These receipts are signed "E. H. GIBBS & Co.," and any which are not so signed are fraudulent. We are always glad to correct errors, but we must ask all persons who deem themselves aggrieved by us, to examine their receipts, and thus learn who is responsible for the shortcomings of which they complain.

